# Leukocyte immunoglobulin‐like receptor B2 regulates atherosclerosis progression by modulating macrophage extracellular trap formation in foam macrophages through the PI3K‐AKT signaling pathway

**DOI:** 10.1002/ccs3.70053

**Published:** 2025-11-11

**Authors:** Yuanyong Jiao, Rui Hu, Liang Gui, Suyu Miu, Xiwei Zhang, Junjie Zou

**Affiliations:** ^1^ Department of Vascular Surgery The First Affiliated Hospital with Nanjing Medical University (Jiangsu Province Hospital) Nanjing China

**Keywords:** atherosclerosis, foam macrophages, LILRB2, METs, PI3K‐AKT signaling pathway

## Abstract

This study aimed to examine the regulatory role of leukocyte immunoglobulin‐like receptor B2 (LILRB2) in macrophage extracellular trap (MET) formation in foam macrophages in atherosclerosis (AS). Three datasets were subjected to bioinformatics analysis to identify differentially expressed genes (DEGs). Atherosclerotic lesions from patients with AS were subjected to hematoxylin and eosin and oil red O staining. The levels of lipid regulation‐related proteins and inflammatory factors were measured in the lesions. MET formation was induced in oxidized low‐density lipoprotein‐treated foam macrophages with tumor necrosis factor‐alpha (TNF‐α). *LILRB2* knockdown cells were established to evaluate the role of LILRB2 in MET formation. In a rat AS model, the levels of PI3K/AKT signaling pathway‐related molecules and METs were measured in groups classified based on LILRB2 expression. *LILRB2* was a key DEG in foam macrophages in AS. The atherosclerotic tissues exhibited increased levels of lipid accumulation and METs and dysregulation of lipid‐related and inflammatory factors. Treatment with TNF‐α promoted MET formation and LILRB2 expression. Y‐P 740 treatment mitigated the *LILRB2* knockdown‐induced suppression of PI3K/AKT signaling and MET formation. LILRB2 mediated AS pathogenesis by promoting MET formation in foam macrophages via the PI3K/AKT pathway. Targeting LILRB2 and its associated signaling pathway was a potential novel therapeutic strategy for AS.

## Graphical abstract

Our research examined how leukocyte immunoglobulin‐like receptor B2 (LILRB2) impacts macrophage extracellular trap (MET) formation in foam macrophages within atherosclerosis (AS). Results highlighted LILRB2's key role in AS, suggesting it may influence PI3K/AKT signaling to spur MET formation. This implies that targeting LILRB2 and related pathways could offer a novel therapeutic approach for AS.

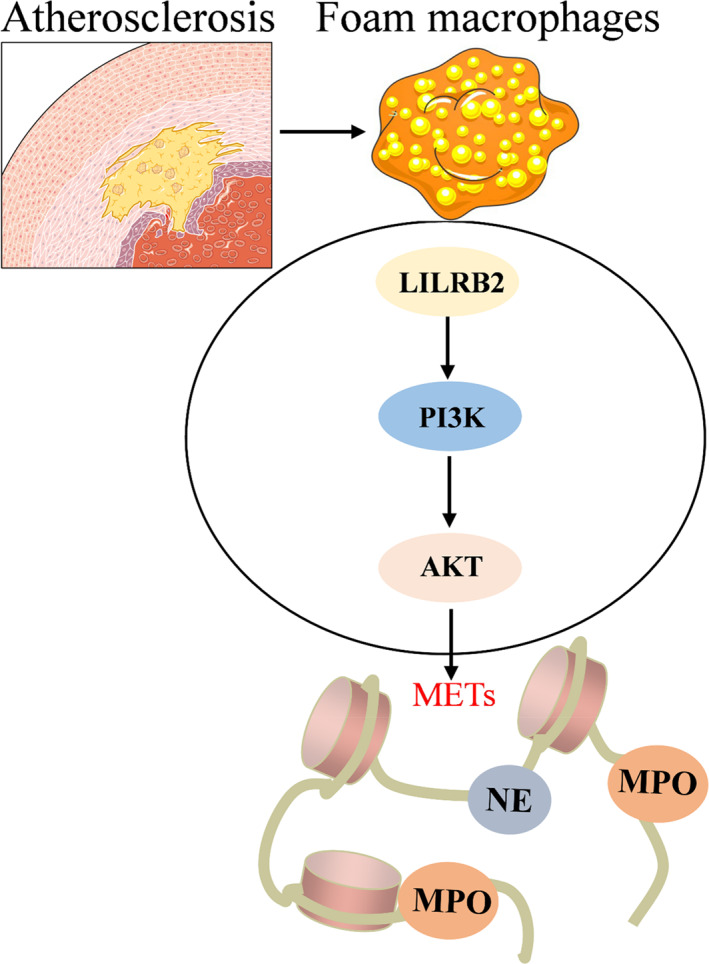

## 1 BACKGROUND

Atherosclerosis (AS) is the main cause of cardiovascular diseases, such as stroke and heart disease.^1^, ^2^ Various immune cells, such as dendritic cells, lymphocytes, macrophages, and neutrophils, are involved in the development of AS. These immune cells dysregulate endothelial cell functions through the activation of adhesion molecules and inflammatory cytokines, contributing to the formation of atherosclerotic plaques.^3^ Macrophages are primarily derived from myeloid progenitor cells in the bone marrow, which mature into circulating monocytes.^4^ After being recruited at the injury site, these monocytes adhere to endothelial cells in the arterial lumen and migrate,^5^ transforming into macrophages in the subcutaneous space.^6^ The increased uptake of oxidized low‐density lipoprotein (ox‐LDL) transforms pro‐inflammatory macrophages into foam cells. The accumulation of foam cells in the developing atherosclerotic lesions enhances inflammation.^7^, ^8^ Thus, foam macrophages have a crucial role in the progression of AS.

Immune cells release extracellular traps (ETs) containing histones, double‐stranded DNA (dsDNA), and elastases to trap and eliminate microorganisms, such as parasites, fungi, and bacteria.^9^ Pathogens and sterile inflammatory stimuli promote the formation of neutrophil ETs (NETs).^10^, ^11^ Clinical and animal studies have demonstrated that NETs are involved in AS development.^12^, ^13^, ^14^ Previous studies have demonstrated that phorbol 12‐myristate 13‐acetate (PMA), hypochlorous acid, *Staphylococcus aureus*, interleukin (IL)‐8, and tumor necrosis factor (TNF)‐α promote the formation of macrophage ETs (METs).^15^, ^16^ However, the mechanism and roles of foam macrophages in MET formation in AS have not been elucidated.

In this study, the following three AS‐related differentially expressed genes (DEGs) were identified in foam macrophages using bioinformatics analysis: four and a half LIM domains protein 1 (*FHL1*), leukocyte immunoglobulin‐like receptor B2 (*LILRB2*), and serpin family A member 1 (*SERPINA1*). The expression levels of *LILRB2* and *SERPINA1* were upregulated, whereas those of *FHL1* were downregulated. This study focused on the potential roles of upregulated genes *LILRB2* in foam macrophages. SERPINA1 is primarily produced in the liver and inactivates proteases, such as neutrophil elastase.^17^ Elastase is one of the main components of METs. This study aimed to identify a gene involved in MET formation. Thus, the regulatory effects of *LILRB2* on MET formation were examined in foam macrophages.

LILRB2, an inhibitory receptor of the immunoglobulin superfamily, is predominantly expressed in myeloid cells, such as granulocytes, monocytes, and macrophages.^18^, ^19^ The mRNA levels of *LILRB2* are upregulated in patients with acute myocardial infarction (AMI).^20^ Additionally, LILRB2 is reported to mediate the development of excessive inflammation in systemic lupus erythematosus, which may lead to the early onset of AS.^21^ Thus, LILRB2 plays an important role in the progression of AS. The knockdown of *LILRB2* impaired the LIR‐A3‐mediated release of NET in NB4 cells.^22^ Six NETosis‐associated genes were identified in anti‐neutrophil cytoplasmic antibody‐associated glomerulonephritis. Analysis of the correlation of these genes with immune cells revealed that macrophages exhibited enhanced expression of these six genes when compared with monocytes and neutrophils.^23^ This suggests that LILRB2 may influence macrophages to release ETs. Therefore, LILRB2 was hypothesized to regulate the formation and release of METs in foam macrophages in AS.

In this study, Kyoto encyclopedia of genes and genomes (KEGG) pathway enrichment analysis revealed that LILRB2 is enriched in the PI3K‐AKT signaling pathway. Previous studies have reported that LILRB2 regulates AKT phosphorylation in fibroblast‐like synovial cells and liver macrophages.^24^, ^25^ AKT is involved in the release of ETs.^26^ In particular, the phosphorylation of AKT and p38‐MAPK, rather than that of ERK1/2, is associated with myeloperoxidase (MPO) secretion.^27^ These findings indicate that LILRB2 can potentially stimulate the formation of METs in foam macrophages by regulating the PI3K/AKT signaling pathway.

This study examined whether LILRB2 regulates the formation of METs in foam macrophages through the PI3K/AKT pathway using cell culture and animal models.

## 2 METHODS

### 2.1 Data collection

Three datasets (GSE159677, GSE9874, and GSE100927) were downloaded from the Gene Expression Omnibus database. The GSE159677 dataset comprises the chromatin immunoprecipitation data of 3 carotid artery proximal adjacent samples and 3 calcified atherosclerotic cores. In this study, three single‐cell samples of AS were selected for analysis. The GSE9874 dataset comprises the data of 60 samples (foam cells and baseline macrophages from subjects with or without AS). All four types of samples were used for subsequent analyses. The GSE100927 dataset comprises the data of 104 samples (atherosclerotic lesion samples and control samples without atherosclerotic lesions), which were all used for subsequent studies.

### 2.2 Single‐cell data analysis

The R package Seurat was used to analyze nFeature (the number of genes), nCount (the number of sequencing reads), and percent.mt (mitochondrial gene content). The filtering criteria were as follows: nFeature_RNA > 500, nCount_RNA > 1000, nFeature_RNA < 7000, nCount_RNA < 70,000, and percent.mt < 30. The sample data were integrated into the R software package “Seurat” using the anchors method to identify core cells. The single‐cell RNA sequencing data were filtered based on the following criteria: expression of genes in ≤3 cells; cells in which <200 genes were expressed. Gene expression in the core cells was normalized using the linear regression model. Next, the single‐cell samples were subjected to principal component analysis (PCA). The top 20 principal components (PCs) were selected for further examination. Overall dimensionality reduction analysis of the first 20 PC sample pairs was performed using the uniform manifold approximation and projection (UMAP) algorithm. To provide additional annotation, the “singleR” package in R was used with Human Primary Cell Atlas Data, Blueprint Encode Data, and Immune Cell Expression Data serving as the reference data.

The Find All Markers function in the Seurat package was used to identify the marker genes for each cluster based on the following parameters: min.pct = 0.2 and only.pos = TRUE. Significant DEGs were identified using the Wilcoxon rank sum test during the screening of marker genes. The cells exhibiting significantly different marker gene expression levels (*p* < 0.05) in AS were classified as core cells.

### 2.3 Identification of DEGs in GSE9874 and GSE100927 datasets

DEGs in the GSE9874 and GSE100927 datasets were identified using the Rlimma package. Briefly, the data were normalized using the normalizeBetweenArrays function. Next, the probe ID was converted into the official genetic symbol. The fold changes and adjusted *p*‐values of each gene were calculated using the lmFit and eBayes functions, respectively. The criteria for identifying DEGs were as follows: adjusted *p* < 0.05; |log fold‐change| > 0.32. The volcano plot and heatmap of the DEGs were generated using the Rggplot2 and heatmap packages, respectively.

### 2.4 Functional enrichment analysis

KEGG pathway and gene ontology (GO) enrichment analyses were performed using the R packages clusterProfiler and org.Hs.eg.db. The enrichments were considered significant at *p* < 0.05.

### 2.5 Patients

The arterial tissues were collected from patients at The First Affiliated Hospital with Nanjing Medical University (Jiangsu Province Hospital). The relevant information of the arterial tissues was presented in Table 1. The study protocol was reviewed and approved by the Ethics Committee of The First Affiliated Hospital with Nanjing Medical University (Jiangsu Province Hospital). This study was performed according to the guidelines of the Declaration of Helsinki. Informed consent was obtained from all the patients.

**TABLE 1:** The information regarding the human arterial tissues.

Groups	Gender	Age	Tissue source
Normal	Woman	52	Superficial femoral artery
	Man	52	Brachial artery
AS	Woman	71	Superficial femoral artery
	Woman	67	Superficial femoral artery
	Man	73	Superficial femoral artery
	Man	62	Superficial femoral artery
	Man	61	Superficial femoral artery

Abbreviation: AS, atherosclerosis.

### 2.6 H&E staining

The arterial tissues were subjected to hematoxylin and eosin (H&E) staining for pathological examination. The tissues were immersed in paraffin wax and sliced into 4‐μm‐thick sections. The sections were dewaxed using xylene, rehydrated using alcohol, and stained with Harris hematoxylin (G1004, Servicebio) and eosin dye (G1002, Servicebio). The stained sections were then dehydrated, sealed with neutral gum, and imaged under a microscope.

### 2.7 Cell line and culture

The human monocytic leukemia cell line (THP‐1 cells; RRID: CVCL_0006), which was obtained from the peripheral blood of a male patient with acute monocytic leukemia aged 1 year, was purchased from Procell (CL‐0233) in 2023. The cell line was authenticated for the described experiments and has not been previously reported as misidentified or contaminated. Additionally, the THP‐1 cell line was determined to be free of mycoplasma contamination.

The THP‐1 cells and THP‐1‐derived macrophage cells were cultured in 1640 medium (GNM31800, GENOM) supplemented with 10% exosome‐depleted fetal bovine serum (FBS) and penicillin–streptomycin solution (GNM15140‐1, GENOM) at 37°C and 5% CO_2_ in a humidified atmosphere. The cells were incubated with PMA for 48 h to promote differentiation into macrophages. Meanwhile, the cells were incubated with ox‐LDL (50 μg/mL) and TNF‐α (50 ng/mL) to induce the formation of foam macrophages and METs, respectively.

### 2.8 Oil red O staining

The arterial tissue sections were stained with a 0.5% oil red O‐isopropanol solution (G1015, Servicebio) for 1 h at 37°C and differentiated with a 75% ethanol solution for 1 min. After washing thrice with phosphate‐buffered saline (PBS), the cell nuclei were stained with hematoxylin for 2 min. The stained sections were then sealed with neutral gelatin and imaged under a microscope.

To perform oil red O staining, cells were washed twice with PBS and fixed with 4% paraformaldehyde for 30 min. Next, the cells were washed twice with PBS and stained with a freshly diluted oil red O solution for 30 min. Finally, the samples were rinsed in PBS and observed under a light microscope.

### 2.9 Western blotting

Total proteins were extracted from the cells or the tissue ground in liquid nitrogen using the cell lysis solution (P0013, Beyotime). The extracted proteins were quantified using the bicinchoninic acid protein assay kit (P0010, Beyotime). The protein samples were subjected to sodium dodecyl sulfate‐polyacrylamide gel electrophoresis. The resolved proteins were transferred to a membrane. The membrane was blocked with 5% non‐fat milk for 2 h and incubated with the following primary antibodies at 4°C overnight: anti‐low‐density lipoprotein receptor (LDL‐R, 1:750, ab52818, Abcam), anti‐SREBP‐1C (1:2000, 14088‐1‐AP, Proteintech), anti‐ABCA1 (1:1000, 26564‐1‐AP, Proteintech), anti‐ABCG1 (1:2000, 13578‐1‐AP, Proteintech), anti‐IL‐6 (1:1000, 21865‐1‐AP, Proteintech), anti‐IL‐8 (1:1000, 27095‐1‐AP, Proteintech), anti‐TNF‐α (1:500, AF7014, Affinity), anti‐LILRB2 (1:1000, 14154‐1‐AP, Proteintech), anti‐p‐PI3K (1:1000, 17366, CST), anti‐PI3K (1:1000, 4292, CST), anti‐p‐AKT (1:2000, p‐AKT, CST), anti‐AKT (1:1000, 9272, CST), and anti‐GAPDH (1:5000, AB0037, Abways) antibodies. Next, the membrane was incubated with horseradish peroxidase (HRP)‐conjugated goat anti‐mouse (Beyotime, A0216) or HRP‐conjugated goat anti‐rabbit (Beyotime, A0208) antibodies. GAPDH was used as the loading control.

### 2.10 Immunofluorescence analysis

The suspensions of THP‐1 cells (5 × 10^5^ cells/mL) were transferred to 24‐well plates and cultured for 48 h. The cells were fixed with 4% paraformaldehyde for 10 min and permeabilized with 0.2% TritonX‐100 in PBS for 10 min. After washing twice with PBS, the cells were blocked with 5% FBS in PBS for 1 h to mitigate non‐specific antibody binding and incubated overnight at 4°C with the anti‐CD68 antibodies (1:200, DF7518, Affinity). Next, the samples were incubated with the secondary antibody (1:500, A0423, Beyotime), mounted with an anti‐fluorescence quenching sealing solution containing 4′,6‐diamidino‐2‐phenylindole (DAPI), and observed under a light microscope.

METs were quantified using immunofluorescence staining after exposure to different conditions. A solution containing SYTOX Green (200 nM) and Hoechst 33342 (10 μg/mL) in PBS was prepared. The samples were incubated with the prepared solution (200 μL) in the culture medium for 10 min. After washing with PBS, the samples were imaged under a microscope.

METs in the paraffin sections of the animal arterial tissues were quantified. The sections were dewaxed, hydrated, subjected to antigen retrieval, blocked with bovine serum protein, and incubated with anti‐CD68 (1:200, DF7518, Affinity) and anti‐CitH3 (1:2000, ab281584, Abcam) antibodies overnight at 4°C. After washing with PBS containing Tween 20 (PBST), the sections were incubated with AF488‐conjugated goat anti‐rabbit (1:500, A0423, Beyotime) secondary antibodies at room temperature. The sections were washed with PBST and stained with DAPI.

### 2.11 Enzyme‐linked immunosorbent assay

The MPO‐DNA complex levels were measured using the commercially available enzyme‐linked immunosorbent assay (ELISA) kits (ml060524, mlbio), following the manufacturer's instructions. The standard group comprised different concentrations of the standard product (50 μL). The test group was incubated with cell supernatant (50 μL), whereas the blank group comprised untreated samples. The samples were then incubated with 100 μL of antibody at 37°C, washed five times, and sequentially incubated with substrate A (50 μL) and substrate B (50 μL) at 37°C for 15 min in the dark. The reaction was terminated using a 50 μL termination solution. The absorbance of the reaction mixture at 450 nm was measured using a microcoder.

### 2.12 qRT‐PCR analysis

The cells or tissues were ground in liquid nitrogen and lysed with Trizol reagent for extracting RNA. After chloroform treatment, the lysates were transferred to a centrifuge tube and incubated with isopropanol. The concentration of RNA was determined. The isolated RNA was reverse‐transcribed into complementary DNA using HiScript III All‐in‐one RT SuperMix Perfect for qPCR (R333‐01, Vazyme). Quantitative real‐time polymerase chain reaction (qRT‐PCR) analysis was performed using ChamQ Universal SYBR qPCR Master Mix (Q711‐02, Vazyme). The primer sequences were as follows: *LILRB2*: 5′‐CAGACAGGGACCATCCCCAA‐3′ (forward) and 5′‐ACGGTACTCCTGGGCTTCAA‐3′ (reverse); *ACTB*: 5′‐GGCGGCACCACCATGTACCCT‐3′ (forward) and 5′‐AGGGGCCGGACTCGTCATACT‐3′ (reverse). The relative gene expression levels were quantified using the 2^−ΔΔCt^ method. *ACTB* served as an internal control. Each experiment was replicated three times.

### 2.13 *LILRB2* knockdown

Short hairpin RNAs (shRNA) and a non‐targeting RNA (scrambled RNA) were synthesized. The shRNA sequences were as follows: sh‐LILRB2‐1: 5′‐GATCGCATCTTGGATTACACGGATACTCGAGTATCCGTGTAATCCAAGATGCTTTTTG‐3′; sh‐LILRB2‐2: 5′‐GATCCCACTCCGTCTAAGATCAATACTCGAGTATTGATCTTAGACGGAGTGGTTTTTG‐3′; sh‐LILRB2‐3: 5′‐GATCTGGCGGCTTCATTCTGTGTAACTCGAGTTACACAGAATGAAGCCGCCATTTTTG‐3′. The shRNA sequences were cloned into the lentiviral vector JS040 between the BamH I and EcoR I cleavage sites to obtain the interference recombinant plasmid JS039‐sh‐LILRB2. JS039‐sh‐LILRB2 or the corresponding control vector was co‐transfected with lentiviral packaging plasmids into 293T cells. THP‐1 cells (2 × 10^5^ cells/mL) were seeded in 6‐well plates (2 mL/well) and incubated with polybrene (8 μg/mL) and viruses containing sh‐NC and sh‐LILRB2 at a multiplicity of infection of 50 at 37°C for 16 h. The cells were centrifuged, re‐suspended in 1640 complete medium, and cultured in culture plates for 48 h. The supernatant obtained after centrifuging the samples was discarded. Stable transfection colonies were selected using 1640 complete medium containing puromycin (2 μg/mL).

### 2.14 Animal model

Male Sprague–Dawley (SD) rats (*n* = 20; age: 7 weeks; body weight: 320−350 g) were allowed to adapt to the new environment for 1 week. During the experiment, the rats had free access to drinking water and were maintained under ambient temperature and humidity conditions. An SD rat model of AS was established as described previously.^28^, ^29^ The rats were divided into the following groups: normal group (*n* = 6): fed on a standard diet; AD group (*n* = 14), fed a high‐fat Western diet (D12079B, Research Diets). The experimental duration was 20 weeks. All animal experiments were approved by the Ethics Committee of Nanjing Medical University (approval number: 2023‐SR‐471).

### 2.15 Statistical analysis

Data are presented as mean ± standard deviation. All statistical analyses were performed using GraphPad Prism 8.0 (GraphPad Software). Means between two groups were compared using Student's *t*‐tests, whereas those between multiple groups were compared using one‐way analysis of variance. Differences were considered significant at *p* < 0.05.

## 3 RESULTS

### 3.1 Identification of atherosclerotic cells

**FIGURE 1:**
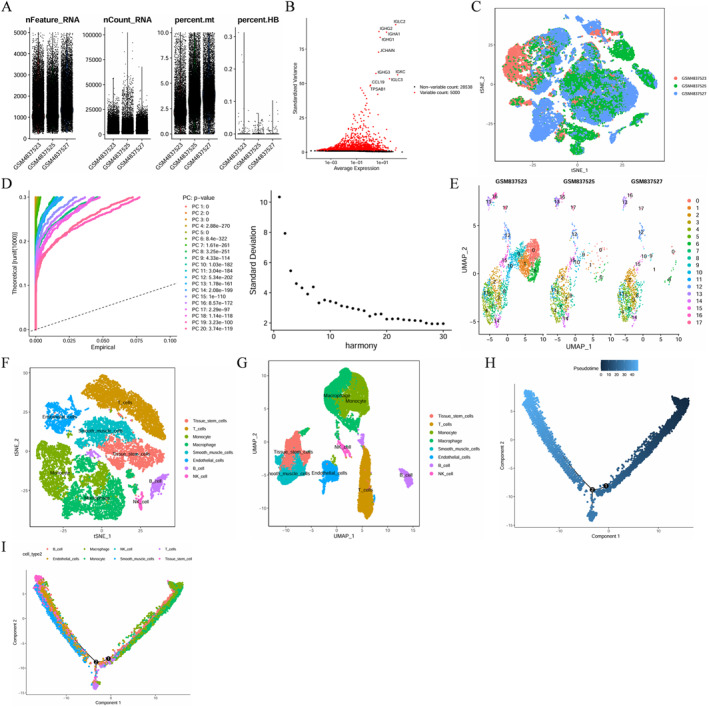
Single‐cell RNA sequencing data analysis of calcified atherosclerotic core samples in the GSE159677 dataset. (A) After filtering the sequencing data, 33,538 core cells were identified. (B) The variance plot illustrates gene expression changes in all atherosclerosis cells (red and black dots indicate highly variable and nonvariable genes, respectively). (C) PCA revealed differential clustering of cells in atherosclerosis. (D) The top 20 PCs (*p* < 0.05) were identified. (E) Cell clusters were classified in each sample. (F, G) All cell clusters in calcified atherosclerosis were annotated with single R and cell markers based on marker gene composition. (H, I) Trajectory analysis revealed two subgroups of atherosclerotic cells with different differentiation patterns. PCA, principal component analysis; PCs, principal components.

After filtering based on the pre‐defined criteria, 33,538 core cells were obtained (Figure 1A). Genetic variation analysis of core cells revealed 5000 genes with high variability (Figure 1B). PCA revealed that the three single‐cell samples exhibited distinct clustering (Figure 1C). Further analysis was performed with 20 PCs (*p* < 0.05) (Figure 1D). The core cells were classified into 18 distinct cell clusters using the UMAP algorithm (Figure 1E) with the “singleR” package and the Cell Marker database. The following eight unique cell clusters were identified: Tissue stem cells, T cells, monocytes, macrophages, smooth muscle cells, endothelial cells, B cells, and natural killer cells (Figure 1F,G).

Through the utilization of Find All Markers and Wilcoxon tests, eight DEGs specific to cells were identified (Table S1). All annotated cells were subjected to pseudo‐temporal analysis using the Monocle 2 algorithm. Atherosclerotic cells exhibited gradual differentiation along two distinct directions (Figure 1H,I).

### 3.2 Identification of DEGs in GSE9874 and GSE100927 datasets

**FIGURE 2:**
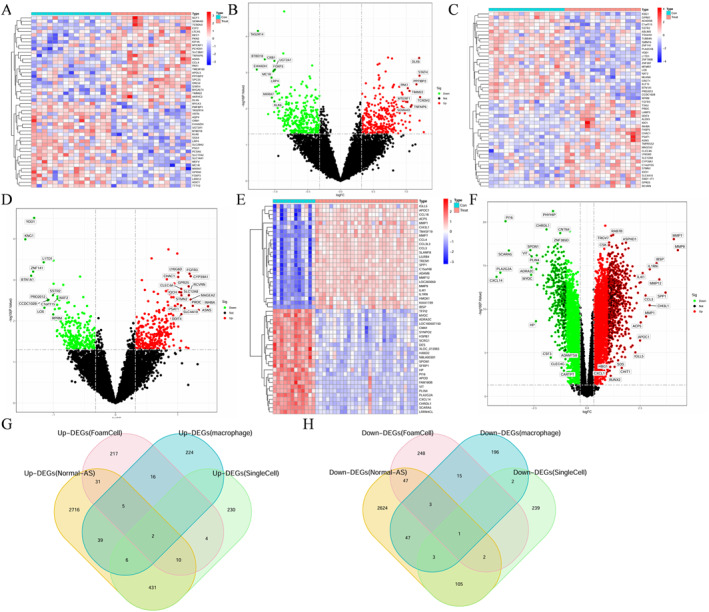
Identification of DEGs in the GSE9874 and GSE100927 datasets. (A) The heatmap of DEGs obtained by comparing the data of foam macrophages in patients with atherosclerosis (AS‐FM group) and those of foam macrophages in patients without atherosclerosis (FM group) in the GSE9874 dataset. (B) The volcano map of DEGs obtained by comparing the AS‐FM and FM groups in the GSE9874 dataset (red and green dots represent upregulated and downregulated genes, respectively). (C) The heatmap of DEGs obtained by comparing the data of baseline macrophages in patients with atherosclerosis (AS‐BM group) and those of baseline macrophages from patients without atherosclerosis (BM group) in the GSE9874 dataset. (D) The volcano map of DEGs obtained by comparing the data of the AS‐BM and BM groups in the GSE9874 dataset (red and green dots represent upregulated and downregulated genes, respectively). (E) The heatmap of DEGs obtained by comparing the data of the AS and Normal groups in the GSE100927 dataset. (F) The volcano map of DEGs obtained by comparing the data of the AS and Normal groups in the GSE100927 dataset (red and green dots represent upregulated and downregulated genes, respectively). (G, H) The common DEGs obtained from single‐cell RNA sequencing data analysis and the DEGs between the following three groups: AS‐FM group versus FM group, AS‐FM group versus FM group, and AS group versus Normal group. AS, atherosclerosis; DEGs, differentially expressed genes.

In the GSE9874 dataset, the data of foam cells from subjects without AS (FM group) and those from subjects with AS (AS‐FM group) were analyzed. Differential analysis revealed 601 DEGs between the AS‐FM and FM groups (Figure 2A,B). Differential analysis of baseline macrophages from subjects without AS (BM group) and those from subjects with AS (AS‐BM group) revealed 559 DEGs (Figure 2C,D). Furthermore, differential analysis of the data of atherosclerotic lesions (AS group) and normal arteries (without atherosclerotic lesions) (normal group) in the GSE100927 dataset revealed 6072 DEGs (Figure 2E,F).

The DEGs between the following three pairs were comparatively examined: AS‐FM group versus FM group; AS‐BM group versus BM group; AS group versus normal group (Figure 2G,H). Three genes related to macrophage foaming lesions involved in the progression of AS were identified (*FHL1*, *LILRB2*, and *SERPINA1*).

### 3.3 Gene expression verification

The expression levels of the core genes in three datasets were verified. In the GSE9874 dataset, the expression levels of the core genes significantly varied between the FM and AS‐FM groups (Figure S1A,B) with area under the curve values exceeding 0.7. In the sample group of “from subjects with AS,” baseline macrophages from both groups also exhibited significant differential expression (Figure S1C,D) with consistent trends. The expression patterns of the three core genes in the GSE100927 dataset were consistent with those in the GSE9874 dataset (Figure S1E,F). Among the three core genes, *LILRB2* and *SERPINA1* were upregulated, whereas *FHL1* was downregulated (Figure S1A,C,E).

### 3.4 Functional enrichment analysis

The median expression levels of LILRB2 in three datasets (AS‐FM, AS‐BM, and AS groups) were noticed. Samples with *LILRB2* expression levels above the median were classified as the high‐expression group, whereas those below the median were classified as the low‐expression group. DEGs between the high‐expression and low‐expression groups were compared. A Venn diagram revealed 48 shared DEGs in any two datasets and all three datasets (Figure S2A). These DEGs were subjected to GO and KEGG pathway enrichment analyses.

LILRB2 was enriched in the following GO terms (Figure S2B): biological process: ossification and extracellular matrix organization; cellular component: collagen‐containing extracellular matrix and external side of plasma membrane; molecular functions: the G protein‐coupled peptide receptor activity and peptide receptor activity.

Meanwhile, LILRB2 was enriched in the following KEGG pathways (Figure S2C): cytokine–cytokine receptor interaction, calcium signaling pathway, and PI3K/AKT signaling pathway.

### 3.5 Histopathological characteristics of vascular tissues in patients with AS

**FIGURE 3:**
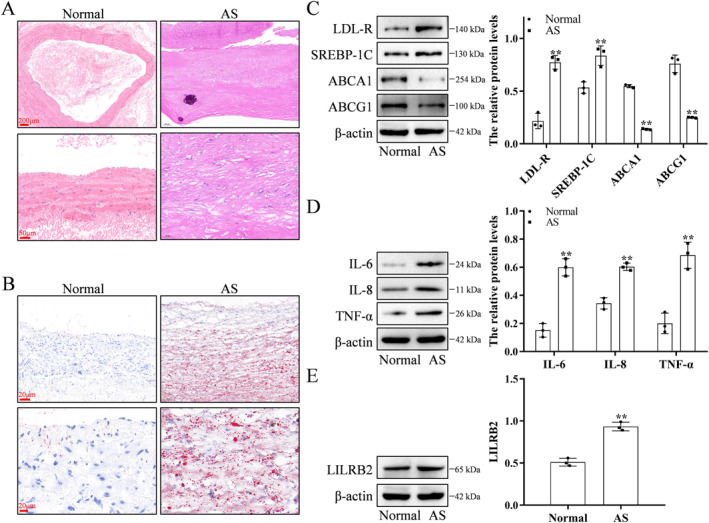
Histopathological characteristics of atherosclerotic and normal artery tissue samples. (A) Hematoxylin and eosin staining of the normal (*n* = 2) and atherosclerotic arterial tissues (*n* = 5). (B) Oil red O staining and hematoxylin and eosin staining of the normal (*n* = 2) and atherosclerotic arterial tissues (*n* = 5). (C) In the atherosclerotic arterial tissues, the levels of LDL‐R and SREBP‐1C were upregulated, whereas those of ABCA1 and ABCG1 were downregulated. (D) The levels of inflammatory factors (IL‐6, IL‐8 and TNF‐α) were upregulated in the atherosclerotic arterial tissues. (E) The expression level of LILRB2 was upregulated in the atherosclerotic arterial tissues. The WB experiment was repeated three times. **p < 0.01, compared with the Normal group. LDL‐R, low‐density lipoprotein receptor; LILRB2, leukocyte immunoglobulin‐like receptor B2; TNF‐α, tumor necrosis factor‐alpha; WB, Western blotting.

The pathological features and the LILRB2 expression levels were examined in the arterial tissues of patients with AS. The arterial tissues from patients with AS exhibited atherosclerotic plaques and lipid accumulation when compared with those from normal patients (Figure 3A,B). The expression levels of LDL‐R and SREBP‐1C were upregulated, whereas those of ABCA1 and ABCG1 were downregulated in the atherosclerotic arterial tissues (Figure 3C). Compared with those in the normal artery tissues, the levels of inflammatory cytokines IL‐6, IL‐8, and TNF‐α were significantly upregulated in the atherosclerotic arterial tissues (Figure 3D). Additionally, LILRB2 expression was upregulated in the atherosclerotic arterial tissues (Figure 3E).

### 3.6 Formation of TNF‐α‐induced METs in macrophages treated with ox‐LDL

**FIGURE 4:**
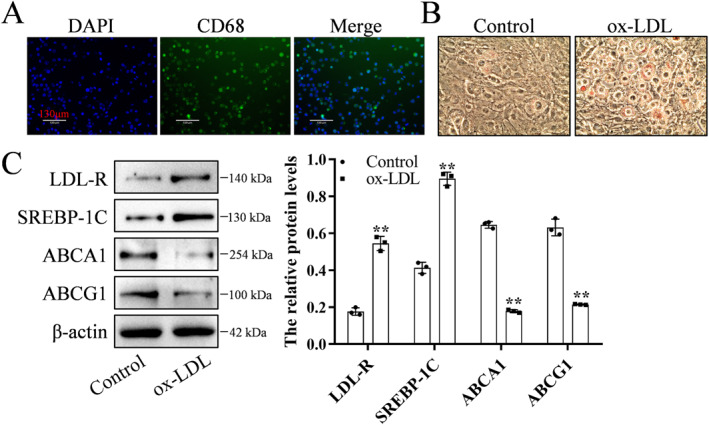
ox‐LDL induces the formation of foam macrophages. (A) Macrophages were characterized using CD86 immunofluorescence staining. The experiment was repeated three times. (B) Macrophages were treated with ox‐LDL and stained with oil red O staining to visualize foam cell formation. The experiment was repeated three times. (C) Treatment with ox‐LDL upregulated the expression levels of LDL‐R and SREBP‐1C and downregulated the levels of ABCA1 and ABCG1. The experiment was repeated three times (*n* = 3). **p < 0.01, compared with the control group. LDL‐R, low‐density lipoprotein receptor; ox‐LDL, oxidized low‐density lipoprotein.

To establish a foam macrophage model, THP‐1 cells were induced to differentiate into macrophages (Figure 4A). Foam macrophages were treated with ox‐LDL, which promoted the accumulation of lipids (Figure 4B). Treatment with ox‐LDL significantly upregulated the expression of LDL‐R and SREBP‐1C and significantly downregulated the expression of ABCA1 and ABCG1 (Figure 4C).

**FIGURE 5:**
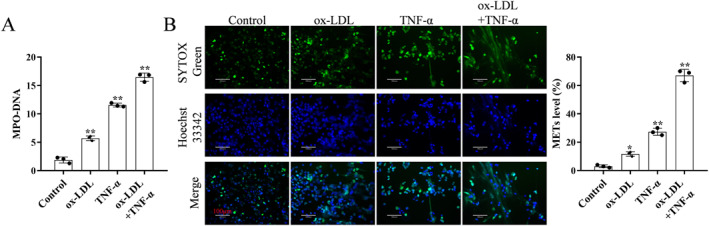
TNF‐α induces the formation of METs in ox‐LDL‐treated macrophages. (A) TNF‐α treatment upregulated the level of MPO‐DNA complex in macrophages treated with ox‐LDL. (B) TNF‐α treatment increased the formation of METs, which were detected using SYTOX Green immunofluorescence staining. The experiment was repeated three times (*n* = 3). *p < 0.05, compared with the control group; **p < 0.01, compared with the control group. METs, macrophage extracellular traps; MPO, myeloperoxidase; ox‐LDL, oxidized low‐density lipoprotein; TNF‐α, tumor necrosis factor‐alpha.

Next, the formation of METs in foam macrophages induced by TNF‐α was examined. Treatment with ox‐LDL or TNF‐α alone increased the concentration of MPO‐DNA. However, the concentration of MPO‐DNA further increased upon treatment with the combination of ox‐LDL and TNF‐α (Figure 5A). Similarly, ox‐LDL and TNF‐α enhanced the formation of METs (Figure 5B).

### 3.7 LILRB2 promotes MET formation by activating the PI3K/AKT signaling pathway

**FIGURE 6:**
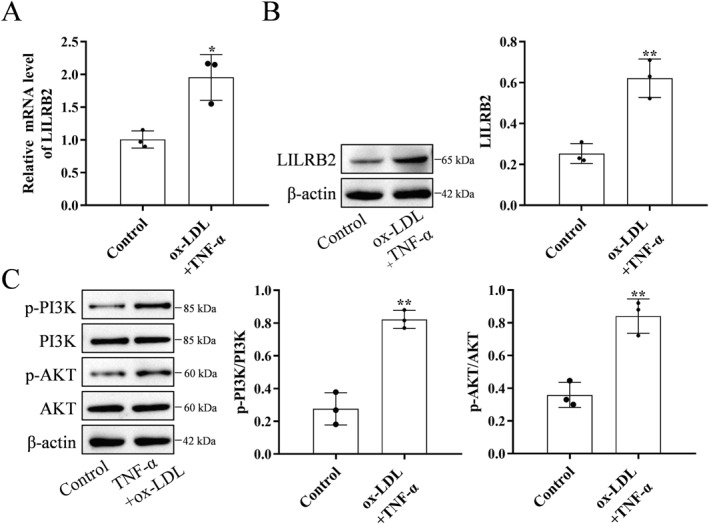
Treatment with TNF‐α and ox‐LDL upregulates LILRB2 and activates PI3K/AKT signaling in foam macrophages. (A) The mRNA level of *LILRB2* was upregulated in TNF‐α and ox‐LDL‐induced macrophages. (B) The protein level of LILRB2 was upregulated in TNF‐α and ox‐LDL‐induced macrophages. (C) The PI3K/AKT signaling pathway was activated in TNF‐α and ox‐LDL‐induced macrophages. The experiment was repeated three times (*n* = 3). *p < 0.05, compared with the control group; **p < 0.01, compared with the control group. LILRB2, leukocyte immunoglobulin‐like receptor B2; ox‐LDL, oxidized low‐density lipoprotein; TNF‐α, tumor necrosis factor‐alpha.

Next, this study examined the role of LILRB2 and its downstream pathway PI3K/AKT in the formation of METs in foam macrophages. Treatment with ox‐LDL and TNF‐α upregulated the mRNA and protein levels of LILRB2 (Figure 6A,B) and the PI3K and AKT phosphorylation levels (Figure 6C) in macrophages.

**FIGURE 7:**
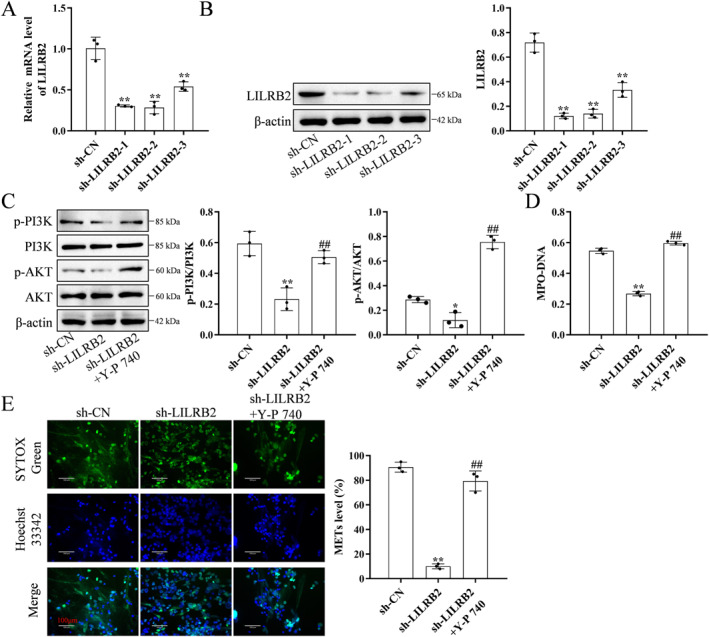
LILRB2 promotes MET formation in TNF‐α and ox‐LDL‐treated macrophages by activating the PI3K/AKT signaling pathway. (A, B) The expression level of LILRB2 was downregulated in *LILRB2* knockdown macrophages. (C) The PI3K/AKT signaling pathway was suppressed in *LILRB2* knockdown macrophages treated with TNF‐α and ox‐LDL. Treatment with Y‐P 740 reversed this effect of *LILRB2* knockdown. (D) The level of MPO‐DNA complex was downregulated in *LILRB2* knockdown macrophages treated with TNF‐α and ox‐LDL. Treatment with Y‐P 740 reversed this effect of *LILRB2* knockdown. (E) Immunofluorescence analysis revealed that the levels of METs were downregulated in *LILRB2* knockdown macrophages treated with TNF‐α and ox‐LDL. Treatment with Y‐P 740 reversed this effect of *LILRB2* knockdown. The experiment was repeated three times (*n* = 3). *p < 0.05, compared with the small interfering RNA negative control (si‐NC)‐transfected group; **p < 0.01, compared with the si‐NC‐transfected group; ^##^ *p* < 0.01, compared with si‐LILRB2‐transfected group. LILRB2, leukocyte immunoglobulin‐like receptor B2; MET, macrophage extracellular trap; MPO, myeloperoxidase; ox‐LDL, oxidized low‐density lipoprotein; si‐LILRB2, LILRB2‐targeting small interfering RNA; si‐NC, small interfering RNA negative control; TNF‐α, tumor necrosis factor‐alpha.

Next, *LILRB2* knockdown macrophages were constructed using si‐LILRB2 (Figure 7A,B). *LILRB2* knockdown downregulated the PI3K and AKT phosphorylation levels. Treatment with the PI3K agonist Y‐P 740 upregulated the PI3K and AKT phosphorylation levels in *LILRB2* knockdown macrophages (Figure 7C). Additionally, Y‐P 740 mitigated the *LILRB2* knockdown‐induced downregulation of MPO‐DNA complex and MET levels (Figure 7D,E).

### 3.8 LILRB2 promotes MET formation in vivo by activating the PI3K/AKT signaling pathway

**FIGURE 8:**
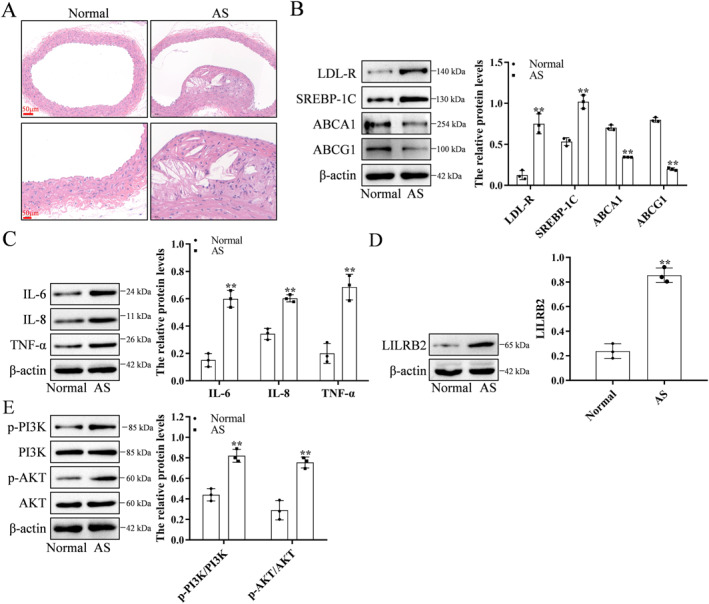
Histopathological characteristics of rat atherosclerotic arterial tissues. (A) Hematoxylin and eosin staining of the Normal (*n* = 5) and atherosclerotic arterial tissues (*n* = 5). (B) Compared with those in the normal arterial tissues, the expression levels of LDL‐R and SREBP‐1C were upregulated and the levels of ABCA1 and ABCG1 were downregulated in the atherosclerotic artery tissues. (C) The levels of inflammatory factors (IL‐6, IL‐8 and TNF‐α) were upregulated in the atherosclerotic artery tissues. (D) The expression level of LILRB2 was upregulated in the atherosclerotic arterial tissues. (E) The PI3K/AKT signaling pathway was activated in the atherosclerotic artery tissues. The WB experiment was repeated three times. **p < 0.01, compared with the Normal group. LDL‐R, low‐density lipoprotein receptor; TNF‐α, tumor necrosis factor‐alpha; WB, Western blotting.

We further constructed an animal model of AS through high‐fat feeding. The arterial tissue exhibited a significant amount of collagen fiber deposition (Figure 8A). In AS artery tissues, the expression levels of LDL‐R and SREBP‐1C were markedly increased, whereas the expression levels of ABCA1 and ABCG1 were notably decreased (Figure 8B). Inflammatory cytokine levels, including IL‐6, IL‐8, and TNF‐α, were significantly higher in atherosclerotic arterial tissues compared to normal arteries (Figure 8C). LILRB2 expression was upregulated in AS artery tissues (Figure 8D). Additionally, phosphorylated PI3K and AKT levels were higher in AS artery tissue (Figure 8E).

**FIGURE 9:**
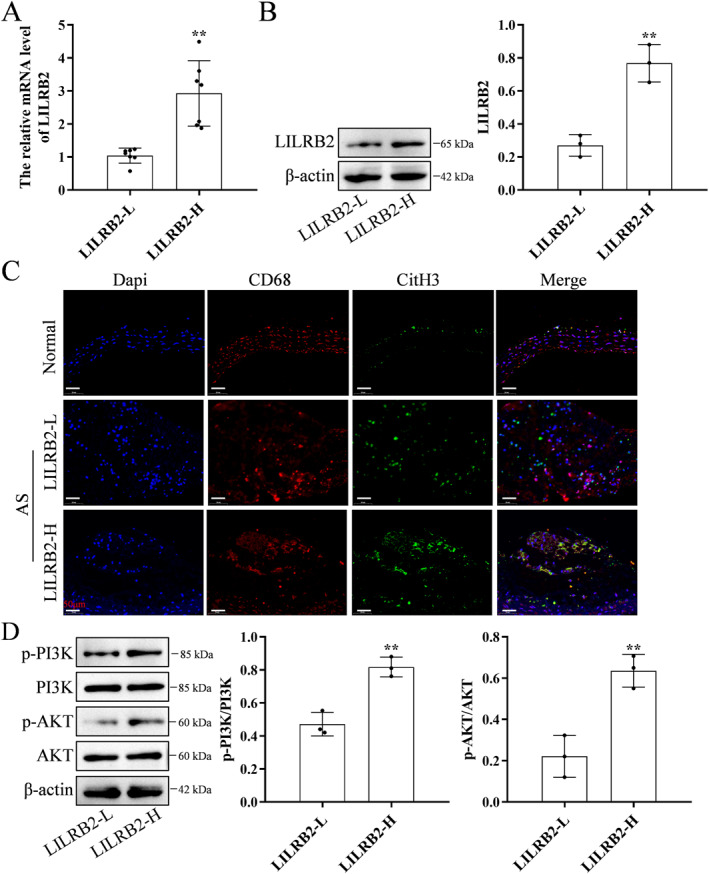
Atherosclerotic arterial tissues exhibiting upregulated LILRB2 expression are associated with enhanced PI3K/AKT signaling pathway activation and MET formation. (A) The mRNA level of *LILRB2* was upregulated in the atherosclerotic artery tissues (*n* = 7) with enhanced LILRB2 expression. (B) The protein level of LILRB2 was upregulated in the atherosclerotic artery tissues with enhanced LILRB2 expression. (C) The levels of METs were determined using CD68, CitH3, and MPO immunostaining. MET formation was upregulated in the atherosclerotic artery tissues (*n* = 3) with enhanced LILRB2 expression. (D) The PI3K/AKT signaling pathway was activated in the atherosclerotic artery tissues with enhanced LILRB2 expression. **p < 0.01, compared with the LILRB2‐low group. LILRB2, leukocyte immunoglobulin‐like receptor B2; MET, macrophage extracellular trap; MPO, myeloperoxidase.

The AS animal models were divided into low‐LILRB2 and high‐LILRB2 expression groups (Figure 9A,B). The levels of downstream signaling pathway‐related molecules and MET formation were evaluated in these groups. The formation of METs (Figure 9C) and the phosphorylation levels of PI3K and AKT were upregulated in the high‐LILRB2 expression group (Figure 9D).

## 4 DISCUSSION

Previous studies have reported that the LILRB2 ligand ANGPTL2 is associated with cardiovascular diseases in humans.^30^, ^31^, ^32^ Analysis of clinical atherosclerotic samples revealed that *LILRB2* is one of the hub genes that is upregulated in atherosclerotic plaques.^31^ Consistently, this study demonstrated that *LILRB2* is upregulated in foam macrophages. Additionally, PirB is reported to be upregulated in the AS mouse model. The expression of LILRB2 in human macrophages has been previously examined.^33^, ^34^ However, the specific molecular mechanisms of LILRB2 in AS have not been elucidated.

Studies on METs are limited and mostly focus on infections^35^, ^36^, ^37^ and autoimmune diseases.^38^, ^39^ Patients with severe coronary AS exhibit high levels of MPO‐DNA complexes, nucleosomes, and circulating dsDNA.^14^ To investigate the effect of LILRB2 on MET formation in foam macrophages, macrophages were induced to differentiate into foam cells using ox‐LDL. Treatment with ox‐LDL promoted the accumulation of large lipids. Previous studies have reported that ox‐LDL promotes the development of AS.^40^ Macrophages engulf ox‐LDL and transform into foam cells. The resulting disruption of lipid metabolism promotes lipid accumulation in macrophages, contributing to the development of atherosclerotic plaques.^41^

In this study, the expression levels of LDL‐R and SREBP‐1C were significantly upregulated, whereas those of ABCA1 and ABCG1 were downregulated in clinical and animal tissue samples and ox‐LDL‐induced macrophages. These molecules regulate the development of foam macrophages by modulating lipid metabolism. Monocyte‐derived macrophages expressing LDL‐R can regulate LDL levels in the blood. The downregulation of LDL‐R can prevent excessive storage of lipids inside cells.^42^ LDL‐R binds to apolipoproteins on LDL, promoting LDL endocytosis.^43^ Macrophages break down cholesterol esters into free cholesterol in lysosomes after phagocytosing LDL particles. Subsequently, cholesterol undergoes re‐esterification in the endoplasmic reticulum, resulting in the buildup of cholesterol esters and the formation of lipid droplets.^44^, ^45^, ^46^ Cholesterol ester is broken down to release cholesterol, which is transported by cholesterol effector transporters, such as ABCA1 and ABCG1 to regulate cholesterol levels in the cell.^44^ SREBF1 primarily regulates the metabolism of fatty acids by activating genes involved in the synthesis of fatty acids and triglycerides.^47^

In this study, TNF‐α stimulated the formation of METs in foam macrophages and upregulated LILRB2 expression. However, *LILRB2* knockdown suppressed the TNF‐α‐induced MET formation in foam macrophages. TNF‐α is reported to promote MET formation in macrophages.^16^ METs in the breast adipose tissue of obese individuals are stimulated by TNF‐α released from dead fat cells.^48^ In addition to TNF‐α, various molecules and factors induce MET formation. For example, *Mannheimia olytica* and *Histophilus somni* can trigger MET formation in bovine macrophages.^49^, ^50^ Furthermore, RAW 264.7 macrophage and human THP‐1‐derived macrophage cell lines generate METs upon exposure to *Escherichia coli* toxins.^49^ Stimulation with *S. aureus* and PMA induces the production of METs in the mouse peritoneal macrophages and RAW 264.7 cell line.^15^ One study reported that macrophages can generate METs from different sources upon exposure to several microbial and external stimuli, such as hypochlorous acid, PMA, and IL‐8.^16^

Although there is limited literature on the influence of LIRB2 on METs formation in macrophages, previous studies have examined its impact on NET formation. *LILRB2* knockdown suppressed NET formation in NB4 cells.^22^ One study examined the gene expression profile of patients undergoing hemodialysis (HD) and those with AMI. NET was the potential link between HD and AMI with *LILRB2* serving as the key gene.^51^ Nine NET‐associated genes, including *LILRB2*, *MMP9*, and *TLR2*, were confirmed to serve as novel biomarkers for AMI. These genes may be involved in the progression of AMI by promoting the formation of NETs.^52^

The AKT signaling pathway is involved in the formation of METs. AKT activates the NADPH oxidase complex and promotes the release of MPO.^53^ Additionally, the phosphorylation of AKT and p38‐MAPK, rather than that of ERK1/2, promotes MPO cell secretion.^27^ In this study, LILRB2/PirB promoted MET formation by activating the PI3K/AKT pathway. LILRB2/PirB also regulates AKT signaling in macrophages. The inhibition of PirB with an antibody can prevent ANGPTL8‐induced AKT phosphorylation in macrophages.^25^ Meanwhile, the inhibition of LILRB2 disrupts AKT activation, potentially interfering with the Sema6D/PI3Kγ/mTOR pathway.^33^

The impact of LILRB2 and PI3K/AKT signaling pathways on the polarization of M1 and M2 macrophages was not investigated in this study. M1 macrophages are reported to be involved in plaque initiation, progression, and instability.^54^, ^55^ Meanwhile, M2 macrophages alleviate inflammation, promote plaque stability, and prevent AS.^56^ The interaction between LILRB2 and ANGPTL8 promotes the pro‐inflammatory transformation of liver macrophages by upregulating AKT phosphorylation, which leads to increased lipid accumulation in hepatocytes.^25^ ANGPTL8 binds to LILRB2/PIRB and promotes the M2 differentiation of macrophages.^57^ Future studies should explore the effects of the LILRB2/PI3K/AKT signaling pathway on macrophage polarization. This study also did not examine the effect of LILRB2 on MET formation in M1 and M2 macrophages. M1 macrophages are reported to generate METs upon stimulation. In vitro studies have demonstrated that M1‐like macrophages, but not M2‐like macrophages, produce METs.^58^, ^59^ M1 macrophages release extracellular DNA in the presence of NETs, while M2 macrophages are involved in NET clearance.^58^ This indicates that the phenotype may determine the ability of macrophages to produce ETs. Future studies should investigate the regulatory effects of LILRB2 on MET formation in M1 macrophages.

This study has some limitations. The atherosclerotic samples from the AS rat model were divided into high‐LILRB2 expression and low‐LILRB2 expression groups to explore the effect of LILRB2 on the formation of METs. Future studies must establish knockout mice to target LILRB2 in macrophages. Moreover, the number of animals used in this study was small and must be increased further to conclusively demonstrate that LILRB2 promotes the formation of METs in AS.

## 5 CONCLUSIONS

This study demonstrated that LILRB2 suppresses the formation of METs in macrophages by regulating the PI3K/AKT signaling pathway. The findings of this study improved our understanding of the molecular mechanisms underlying AS pathogenesis. Additionally, these findings suggest that LILRB2 is a potential therapeutic target for AS.

## AUTHOR CONTRIBUTIONS

Xiwei Zhang and Junjie Zou contributed to the study conception and design. Material preparation, data collection, and analysis were performed by Yuanyong Jiao, Rui Hu, Liang Gui, and Suyu Miu. Liang Gui and Suyu Miu contributed to literature search and data visualization. The first draft of the manuscript was written by Yuanyong Jiao. All authors read and approved the final manuscript.

## CONFLICT OF INTEREST STATEMENT

The authors declare no conflicts of interest.

## ETHICS STATEMENT

The study protocol was reviewed and approved by the Ethics Committee of The First Affiliated Hospital with Nanjing Medical University (Jiangsu Province Hospital) in accordance with the Declaration of Helsinki. All animal experiments were approved by the Ethics Committee of Nanjing Medical University (approval number: 2023‐SR‐471).

## CONSENT TO PARTICIPATE

Written informed consent was taken from all the patients.

## Supporting information

Figures S1 and S2

Tables S1

## Data Availability

The data used to support the findings of this study are available from the corresponding author upon request.

## References

[1] Dahl, A. , C. Lund , and D. Russell . 2007. “[Atherosclerosis and Cerebral Infarction].” Tidsskrift for den Norske laegeforening : tidsskrift for praktisk medicin, ny raekke 127(7): 892–896. 17435812

[2] Malekmohammad, K. , E. E. Bezsonov , and M. Rafieian‐Kopaei . 2021. “Role of Lipid Accumulation and Inflammation in Atherosclerosis: Focus on Molecular and Cellular Mechanisms.” Frontiers in Cardiovascular Medicine 8: 707529. 10.3389/fcvm.2021.707529. 34552965 PMC8450356

[3] Chávez‐Sánchez, L. , J. E. Espinosa‐Luna , K. Chávez‐Rueda , M. V. Legorreta‐Haquet , E. Montoya‐Díaz , and F. Blanco‐Favela . 2014. “Innate Immune System Cells in Atherosclerosis.” Archives of Medical Research 45: 1–14. 10.1016/j.arcmed.2013.11.007. 24326322

[4] Robbins, C. S. , A. Chudnovskiy , P. J. Rauch , J.‐L. Figueiredo , Y. Iwamoto , R. Gorbatov , M. Etzrodt , et al. 2012. “Extramedullary Hematopoiesis Generates Ly‐6C(High) Monocytes that Infiltrate Atherosclerotic Lesions.” Circulation 125(2): 364–374. 10.1161/circulationaha.111.061986. 22144566 PMC3263762

[5] Gerhardt, T. , and K. Ley . 2015. “Monocyte Trafficking Across the Vessel Wall.” Cardiovascular Research 107(3): 321–330. 10.1093/cvr/cvv147. 25990461 PMC4592323

[6] Randolph, G. J. 2014. “Mechanisms that Regulate Macrophage Burden in Atherosclerosis.” Circulation Research 114(11): 1757–1771. 10.1161/circresaha.114.301174. 24855200 PMC4059102

[7] Marchio, P. , S. Guerra‐Ojeda , J. M. Vila , M. Aldasoro , V. M. Victor , and M. D. Mauricio . 2019. “Targeting Early Atherosclerosis: A Focus on Oxidative Stress and Inflammation.” Oxidative Medicine and Cellular Longevity 2019: 8563845. 10.1155/2019/8563845. 31354915 PMC6636482

[8] Sukhorukov, V. N. , V. A. Khotina , Y. S. Chegodaev , E. Ivanova , I. A. Sobenin , and A. N. Orekhov . 2020. “Lipid Metabolism in Macrophages: Focus on Atherosclerosis.” Biomedicines 8: 262. 10.3390/biomedicines8080262. 32752275 PMC7459513

[9] Brinkmann, V. , U. Reichard , C. Goosmann , B. Fauler , Y. Uhlemann , D. S. Weiss , Y. Weinrauch , and A. Zychlinsky . 2004. “Neutrophil Extracellular Traps Kill Bacteria.” Science (New York, NY) 303(5663): 1532–1535. 10.1126/science.1092385. 15001782

[10] Qi, H. , S. Yang , and L. Zhang . 2017. “Neutrophil Extracellular Traps and Endothelial Dysfunction in Atherosclerosis and Thrombosis.” Frontiers in Immunology 8: 928. 10.3389/fimmu.2017.00928. 28824648 PMC5545592

[11] Sørensen, O. E. , and N. Borregaard . 2016. “Neutrophil Extracellular Traps – The Dark Side of Neutrophils.” The Journal of Clinical Investigation 126(5): 1612–1620. 10.1172/jci84538. 27135878 PMC4855925

[12] Knight, J. S. , W. Luo , A. A. O'Dell , S. Yalavarthi , W. Zhao , V. Subramanian , C. Guo , et al. 2014. “Peptidylarginine Deiminase Inhibition Reduces Vascular Damage and Modulates Innate Immune Responses in Murine Models of Atherosclerosis.” Circulation Research 114(6): 947–956. 10.1161/circresaha.114.303312. 24425713 PMC4185401

[13] Warnatsch, A. , M. Ioannou , Q. Wang , and V. Papayannopoulos . 2015. “Inflammation. Neutrophil Extracellular Traps License Macrophages for Cytokine Production in Atherosclerosis.” Science (New York, NY) 349(6245): 316–320. 10.1126/science.aaa8064. PMC4854322 26185250

[14] Borissoff, J. I. , I. A. Joosen , M. O. Versteylen , A. Brill , T. A. Fuchs , A. S. Savchenko , M. Gallant , et al. 2013. “Elevated Levels of Circulating DNA and Chromatin Are Independently Associated With Severe Coronary Atherosclerosis and a Prothrombotic State.” Arteriosclerosis, Thrombosis, and Vascular Biology 33(8): 2032–2040. 10.1161/atvbaha.113.301627. 23818485 PMC3806482

[15] Chow, O. A. , M. von Köckritz‐Blickwede , A. T. Bright , M. E. Hensler , A. S. Zinkernagel , A. L. Cogen , R. L. Gallo , et al. 2010. “Statins Enhance Formation of Phagocyte Extracellular Traps.” Cell Host & Microbe 8(5): 445–454. 10.1016/j.chom.2010.10.005. 21075355 PMC3008410

[16] Rayner, B. S. , Y. Zhang , B. E. Brown , L. Reyes , V. C. Cogger , and C. L. Hawkins . 2018. “Role of Hypochlorous Acid (HOCl) and Other Inflammatory Mediators in the Induction of Macrophage Extracellular Trap Formation.” Free Radical Biology & Medicine 129: 25–34. 10.1016/j.freeradbiomed.2018.09.001. 30189264

[17] King, P. T. , L. Dousha , N. Clarke , J. Schaefer , R. Carzino , R. Sharma , K. L. Wan , et al. 2021. “Phagocyte Extracellular Traps in Children with Neutrophilic Airway Inflammation.” ERJ Open Research 7(2): 00883–02020. 10.1183/23120541.00883-2020. 34164555 PMC8215332

[18] Ravetch, J. V. , and L. L. Lanier . 2000. “Immune Inhibitory Receptors.” Science (New York, NY) 290(5489): 84–89. 10.1126/science.290.5489.84. 11021804

[19] Liang, S. , V. Ristich , H. Arase , J. Dausset , E. D. Carosella , and A. Horuzsko . 2008. “Modulation of Dendritic Cell Differentiation by HLA‐G and ILT4 Requires the IL‐6–STAT3 Signaling Pathway.” Proceedings of the National Academy of Sciences of the United States of America 105(24): 8357–8362. 10.1073/pnas.0803341105. 18550825 PMC2448841

[20] Yan, W. , H. Song , J. Jiang , W. Xu , Z. Gong , Q. Duan , C. Li , Y. Xie , and L. Wang . 2016. “Characteristics of B Cell‐Associated Gene Expression in Patients with Coronary Artery Disease.” Molecular Medicine Reports 13(5): 4113–4121. 10.3892/mmr.2016.5029. 27035867

[21] Zhang, H. , Y. Huang , X. Li , W. Chen , Y. Lun , and J. Zhang . 2023. “Exploration of Hub Genes and Pathogenetic Pathways in Systemic Lupus Erythematosus Complicated with Early Onset Atherosclerosis.” Mediators of Inflammation 2023: 4508436. 10.1155/2023/4508436.

[22] Wang, M. , M. Liu , J. Jia , H. Shi , J. Teng , H. Liu , Y. Sun , et al. 2021. “Association of the Leukocyte Immunoglobulin‐Like Receptor A3 Gene with Neutrophil Activation and Disease Susceptibility in Adult‐Onset Still's disease.” Arthritis & Rheumatology 73(6): 1033–1043. 10.1002/art.41635. 33381895 PMC8252061

[23] Tao, M. , Y. He , L. Li , W. Liao , H. Nie , and P. Gao . 2023. “Identification and Validation of Immune‐Associated NETosis Subtypes and Biomarkers in Anti‐Neutrophil Cytoplasmic Antibody Associated Glomerulonephritis.” Frontiers in Immunology 14: 1177968. 10.3389/fimmu.2023.1177968. 37465687 PMC10351423

[24] Nishiyama, S. , N. Hirose , M. Yanoshita , M. Takano , N. Kubo , Y. Yamauchi , A. Onishi , et al. 2021. “ANGPTL2 Induces Synovial Inflammation via LILRB2.” Inflammation 44(3): 1108–1118. 10.1007/s10753-020-01406-7. 33538932

[25] Li, D. P. , L. Huang , R. R. Kan , X.‐Y. Meng , S.‐Y. Wang , H.‐J. Zou , Y.‐M. Guo , et al. 2023. “LILRB2/PirB Mediates Macrophage Recruitment in Fibrogenesis of Nonalcoholic Steatohepatitis.” Nature Communications 14(1): 4436. 10.1038/s41467-023-40183-3. PMC10363120 37481670

[26] Hosseinzadeh, A. , P. R. Thompson , B. H. Segal , and C. F. Urban . 2016. “Nicotine Induces Neutrophil Extracellular Traps.” Journal of Leukocyte Biology 100(5): 1105–1112. 10.1189/jlb.3ab0815-379rr. 27312847 PMC5069087

[27] Boussif, A. , L. Rolas , E. Weiss , H. Bouriche , R. Moreau , and A. Périanin . 2016. “Impaired Intracellular Signaling, Myeloperoxidase Release and Bactericidal Activity of Neutrophils from Patients with Alcoholic Cirrhosis.” Journal of Hepatology 64(5): 1041–1048. 10.1016/j.jhep.2015.12.005. 26719020

[28] Yue, L. , Z. Fan , H. Xiaomeng , X. U. Ningyang , Z. Yu , W. Qige , W. Jianan , L. U. Bingjiu , and Z. Yan . 2023. “Jianpi Qutan Fang Induces Anti‐Atherosclerosis and Ameliorates Endothelial Cell Injury in High‐Fat Diet Ratsan Anti‐Inflammatory and Inhibiting Janus Kinase/Signal Transducer and Activator of Transcription Signaling Pathway.” Journal of Traditional Chinese Medicine = Chung i tsa chih ying wen pan 43(6): 1168–1175. 10.19852/j.cnki.jtcm.20230814.002. 37946479 PMC10623252

[29] Basu, D. , Y. Hu , L. A. Huggins , A. E. Mullick , M. J. Graham , T. Wietecha , S. Barnhart , et al. 2018. “Novel Reversible Model of Atherosclerosis and Regression Using Oligonucleotide Regulation of the LDL Receptor.” Circulation Research 122(4): 560–567. 10.1161/circresaha.117.311361. 29321129 PMC5815899

[30] Gellen, B. , N. Thorin‐Trescases , P. Sosner , E. Gand , P.‐J. Saulnier , S. Ragot , M. Fraty , et al. 2016. “ANGPTL2 is Associated with an Increased Risk of Cardiovascular Events and Death in Diabetic Patients.” Diabetologia 59(11): 2321–2330. 10.1007/s00125-016-4066-5. 27491833

[31] Meng, Y. , C. Zhang , L. Liang , L. Wei , H. Wang , F. Zhou , R. Li , D. Zou , X. Huang , and J. Liu . 2021. “Identification of Potential Key Genes Involved in the Carotid Atherosclerosis.” Clinical Interventions in Aging 16: 1071–1084. 10.2147/cia.s312941. 34140767 PMC8203271

[32] Tian, Z. , K. Miyata , T. Kadomatsu , H. Horiguchi , H. Fukushima , S. Tohyama , Y. Ujihara , et al. 2016. “ANGPTL2 Activity in Cardiac Pathologies Accelerates Heart Failure by Perturbing Cardiac Function and Energy Metabolism.” Nature Communications 7(1): 13016. 10.1038/ncomms13016. PMC5052800 27677409

[33] Chen, H. M. , W. van der Touw , Y. S. Wang , K. Kang , S. Mai , J. Zhang , D. Alsina‐Beauchamp , et al. 2018. “Blocking Immunoinhibitory Receptor LILRB2 Reprograms Tumor‐Associated Myeloid Cells and Promotes Antitumor Immunity.” The Journal of Clinical Investigation 128(12): 5647–5662. 10.1172/jci97570. 30352428 PMC6264729

[34] van Dalen, F. J. , M. van Stevendaal , F. L. Fennemann , M. Verdoes , and O. Ilina . 2018. “Molecular Repolarisation of Tumour‐Associated Macrophages.” Molecules (Basel, Switzerland) 24(1): 9. 10.3390/molecules24010009. 30577495 PMC6337345

[35] Mónaco, A. , N. Canales‐Huerta , J. Jara‐Wilde , S. Härtel , J. A. Chabalgoity , M. Moreno , and P. Scavone . 2021. “ *Salmonella typhimurium* Triggers Extracellular Traps Release in Murine Macrophages.” Frontiers in Cellular and Infection Microbiology 11: 639768. 10.3389/fcimb.2021.639768. 33981627 PMC8107695

[36] Li, L. , X. Li , G. Li , P. Gong , X. Zhang , Z. Yang , J. Yang , and J. Li . 2018. “Mouse Macrophages Capture and Kill Giardia Lamblia by Means of Releasing Extracellular Trap.” Developmental & Comparative Immunology 88: 206–212. 10.1016/j.dci.2018.07.024. 30048699

[37] Kalsum, S. , C. Braian , V. Koeken , J. Raffetseder , M. Lindroth , R. van Crevel , and M. Lerm . 2017. “The Cording Phenotype of *Mycobacterium tuberculosis* Induces the Formation of Extracellular Traps in Human Macrophages.” Frontiers in Cellular and Infection Microbiology 7: 278. 10.3389/fcimb.2017.00278. 28695112 PMC5483443

[38] Okubo, K. , M. Kurosawa , M. Kamiya , Y. Urano , A. Suzuki , K. Yamamoto , K. Hase , et al. 2018. “Macrophage Extracellular Trap Formation Promoted by Platelet Activation is a Key Mediator of Rhabdomyolysis‐Induced Acute Kidney Injury.” Nature Medicine 24(2): 232–238. 10.1038/nm.4462. 29309057

[39] Allison, S. J. 2018. “Acute Kidney Injury: Macrophage Extracellular Traps in Rhabdomyolysis‐Induced AKI.” Nature Reviews Nephrology 14(3): 141. 10.1038/nrneph.2018.5. 29355172

[40] Leitinger, N. 2005. “Oxidized Phospholipids as Triggers of Inflammation in Atherosclerosis.” Molecular Nutrition & Food Research 49(11): 1063–1071. 10.1002/mnfr.200500086. 16270279

[41] Moore, K. J. , F. J. Sheedy , and E. A. Fisher . 2013. “Macrophages in Atherosclerosis: A Dynamic Balance.” Nature Reviews Immunology 13(10): 709–721. 10.1038/nri3520. PMC4357520 23995626

[42] Matsuura, E. , K. Kobayashi , M. Tabuchi , and L. R. Lopez . 2006. “Oxidative Modification of Low‐Density Lipoprotein and Immune Regulation of Atherosclerosis.” Progress in Lipid Research 45(6): 466–486. 10.1016/j.plipres.2006.05.001. 16790279

[43] Gent, J. , and I. Braakman . 2004. “Low‐Density Lipoprotein Receptor Structure and Folding.” Cellular and Molecular Life Sciences: CMLS 61(19–20): 2461–2470. 10.1007/s00018-004-4090-3. 15526154 PMC11924501

[44] Wang, D. , Y. Yang , Y. Lei , N. T. Tzvetkov , X. Liu , A. W. K. Yeung , S. Xu , and A. G. Atanasov . 2019. “Targeting Foam Cell Formation in Atherosclerosis: Therapeutic Potential of Natural Products.” Pharmacological Reviews 71(4): 596–670. 10.1124/pr.118.017178. 31554644

[45] Tabas, I. , and K. E. Bornfeldt . 2016. “Macrophage Phenotype and Function in Different Stages of Atherosclerosis.” Circulation Research 118(4): 653–667. 10.1161/circresaha.115.306256. 26892964 PMC4762068

[46] Tian, K. , S. Ogura , P. J. Little , S. W. Xu , and T. Sawamura . 2019. “Targeting LOX‐1 in Atherosclerosis and Vasculopathy: Current Knowledge and Future Perspectives.” Annals of the New York Academy of Sciences 1443(1): 34–53. 10.1111/nyas.13984. 30381837

[47] Li, Y. , S. Xu , M. M. Mihaylova , B. Zheng , X. Hou , B. Jiang , O. Park , et al. 2011. “AMPK Phosphorylates and Inhibits SREBP Activity to Attenuate Hepatic Steatosis and Atherosclerosis in Diet‐Induced Insulin‐Resistant Mice.” Cell Metabolism 13(4): 376–388. 10.1016/j.cmet.2011.03.009. 21459323 PMC3086578

[48] Mohanan, S. , S. Horibata , J. L. McElwee , A. J. Dannenberg , and S. A. Coonrod . 2013. “Identification of Macrophage Extracellular Trap‐Like Structures in Mammary Gland Adipose Tissue: A Preliminary Study.” Frontiers in Immunology 4: 67. 10.3389/fimmu.2013.00067. 23508122 PMC3600535

[49] Aulik, N. A. , K. M. Hellenbrand , and C. J. Czuprynski . 2012. “ *Mannheimia haemolytica* and Its Leukotoxin Cause Macrophage Extracellular Trap Formation by Bovine Macrophages.” Infection and Immunity 80(5): 1923–1933. 10.1128/iai.06120-11. 22354029 PMC3347434

[50] Hellenbrand, K. M. , K. M. Forsythe , J. J. Rivera‐Rivas , C. J. Czuprynski , and N. A. Aulik . 2013. “ *Histophilus somni* Causes Extracellular Trap Formation by Bovine Neutrophils and Macrophages.” Microbial Pathogenesis 54: 67–75. 10.1016/j.micpath.2012.09.007. 23022668 PMC7125803

[51] Yang, Y. , Y. Y. Jiao , Z. Zhang , L. Zhuo , and W. G. Li . 2023. “Neutrophil Extracellular Trap is an Important Connection Between Hemodialysis and Acute Myocardial Infarction.” Renal Failure 45(1): 2216307. 10.1080/0886022x.2023.2216307. 37246754 PMC10228304

[52] Cao Y. , Q.‐F. Wang , B. Li , Y.‐L. Zou , and J.‐C. Zhang . 2023. “Identifying Potential Biomarkers in Acute Myocardial Infarction Based on Neutrophil Extracellular Traps Associated Genes.”

[53] Moreau, R. , A. Périanin , and V. Arroyo . 2019. “Review of Defective NADPH Oxidase Activity and Myeloperoxidase Release in Neutrophils from Patients with Cirrhosis.” Frontiers in Immunology 10: 1044. 10.3389/fimmu.2019.01044. 31134093 PMC6517494

[54] Mantovani, A. , C. Garlanda , and M. Locati . 2009. “Macrophage Diversity and Polarization in Atherosclerosis: A Question of Balance.” Arteriosclerosis, Thrombosis, and Vascular Biology 29(10): 1419–1423. 10.1161/atvbaha.108.180497. 19696407

[55] Shalhoub, J. , M. A. Falck‐Hansen , A. H. Davies , and C. Monaco . 2011. “Innate Immunity and Monocyte‐Macrophage Activation in Atherosclerosis.” Journal of Inflammation (London, England) 8(1): 9. 10.1186/1476-9255-8-9. 21526997 PMC3094203

[56] Feig, J. E. , S. Parathath , J. X. Rong , S. L. Mick , Y. Vengrenyuk , L. Grauer , S. G. Young , and E. A. Fisher . 2011. “Reversal of Hyperlipidemia with a Genetic Switch Favorably Affects the Content and Inflammatory State of Macrophages in Atherosclerotic Plaques.” Circulation 123(9): 989–998. 10.1161/circulationaha.110.984146. 21339485 PMC3131163

[57] Gao, Y. , Y. Yuan , S. Wen , Y. Chen , Z. Zhang , Y. Feng , B. Jiang , et al. 2023. “Dual Role of ANGPTL8 in Promoting Tumor Cell Proliferation and Immune Escape During Hepatocarcinogenesis.” Oncogenesis 12(1): 26. 10.1038/s41389-023-00473-3. 37188659 PMC10185523

[58] Nakazawa, D. , H. Shida , Y. Kusunoki , A. Miyoshi , S. Nishio , U. Tomaru , T. Atsumi , and A. Ishizu . 2016. “The Responses of Macrophages in Interaction with Neutrophils that Undergo NETosis.” Journal of Autoimmunity 67: 19–28. 10.1016/j.jaut.2015.08.018. 26347075

[59] Zhang, Y. , B. S. Rayner , M. Jensen , and C. L. Hawkins . 2019. “In Vitro Stimulation and Visualization of Extracellular Trap Release in Differentiated Human Monocyte‐Derived Macrophages.” Journal of Visualized Experiments: JoVE (153): e60541. 10.3791/60541. 31736503

