# Supplementary material for: Leukocyte immunoglobulin‐like receptor B2 regulates atherosclerosis progression by modulating macrophage extracellular trap formation in foam macrophages through the PI3K‐AKT signaling pathway
Source: J Cell Commun Signal. 2025 Nov 11;19(4):e70053. doi: 10.1002/ccs3.70053 (PMC12606016; doi:10.1002/ccs3.70053)
Supplement: Supplementary file 1 — Figures S1 and S2 [file CCS3-19-e70053-s002.doc]

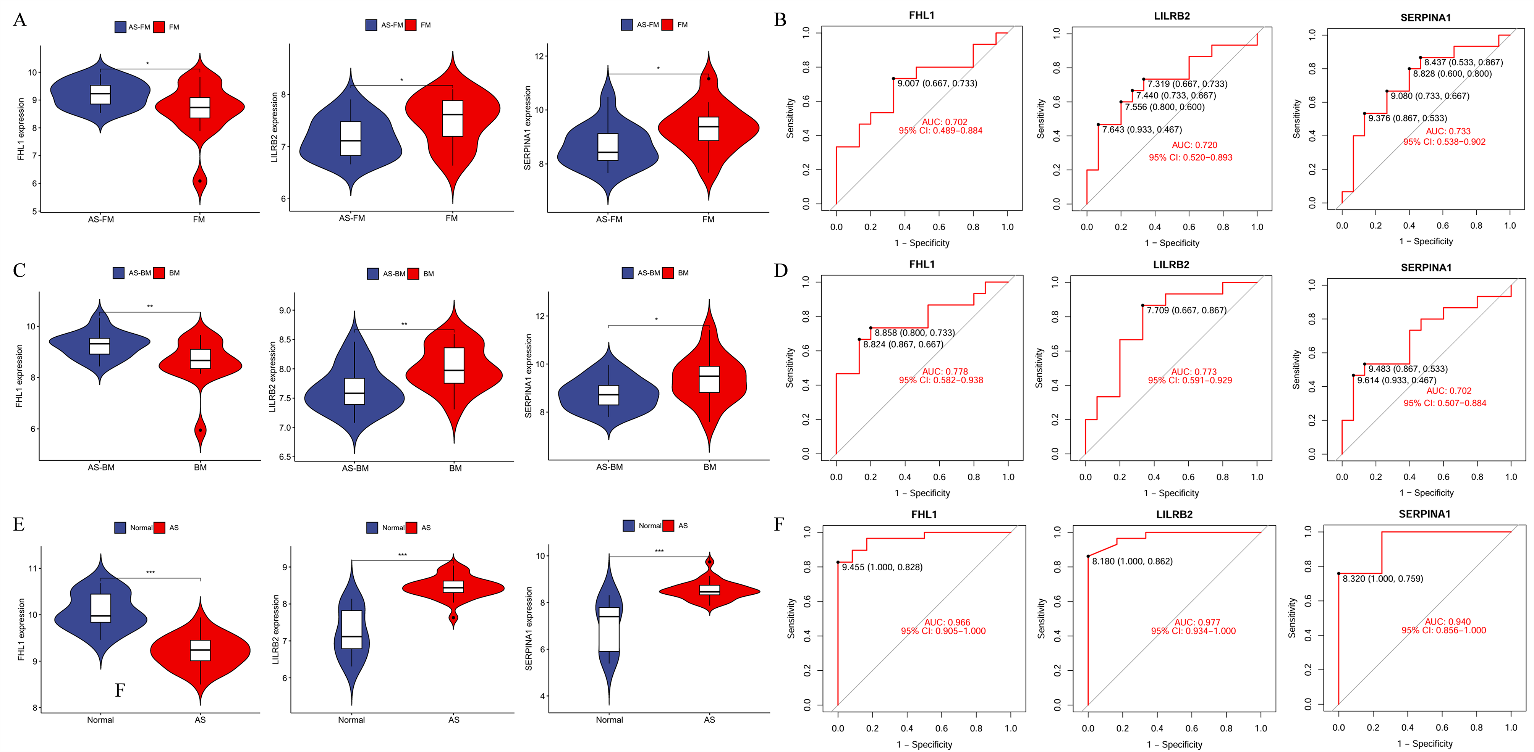


**Figure S1**. Gene expression verification and receiver operating characteristic (ROC) curve analysis. (A, B) The significant differences and the specificity of core genes were verified by comparing the data of foam macrophages from patients with atherosclerosis (AS-FM group) and those from patients without atherosclerosis (FM group) in the GSE9874 dataset. (C, D) The significant differences and the specificity of core genes were verified by comparing the data of baseline macrophages from patients with atherosclerosis (AS-BM group) and those from patients without atherosclerosis (BM group) in the GSE9874 dataset. (E, F) The significant differences and the specificity of core genes were verified by comparing the data of atherosclerotic lesions and Normal arteries in the GSE100927 dataset.


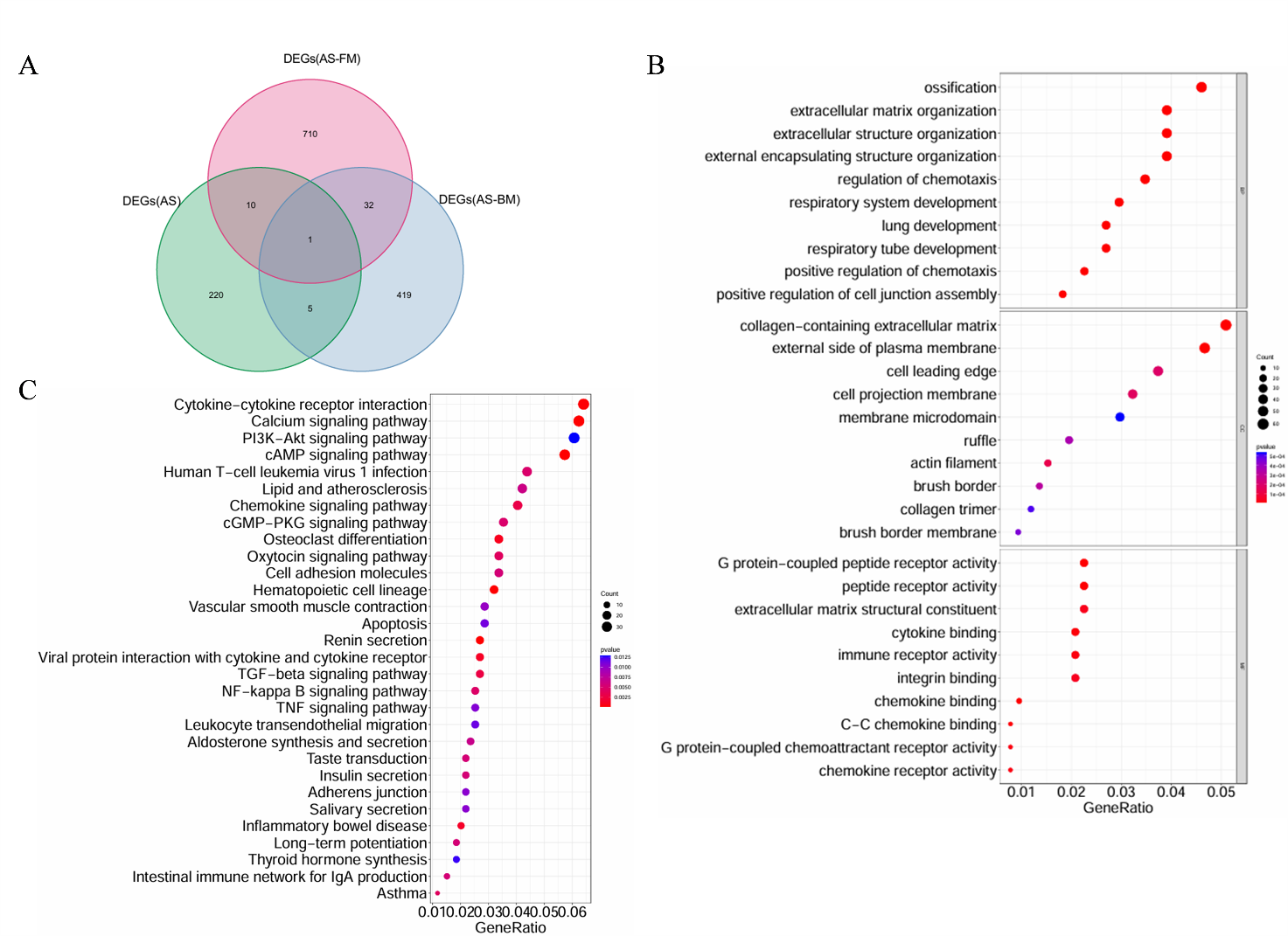


**Figure S2** (A) Comparative analysis of gene expression in foam macrophages from patients with atherosclerosis (AS-FM group), baseline macrophages from patients with atherosclerosis (AS-FM group), and patients with atherosclerotic lesions (AS group) revealed 48 shared differentially expressed genes (DEGs). (B, C) All 48 DEGs were subjected to Gene Ontology (GO) analysis and Kyoto Encyclopedia of Genes and Genomes (KEGG) pathway enrichment analyses.
